# Predictors of Right Ventricular Ejection Fraction in Patients with Nonischemic Cardiomyopathy

**DOI:** 10.1186/1532-429X-18-S1-P297

**Published:** 2016-01-27

**Authors:** Sharif A Sabe, Nisarat Charoensri, Marwa A Sabe, Zoran Popovic, WH Wilson Tang, Scott Flamm, Deborah Kwon

**Affiliations:** Cleveland Clinic Foundation, Cleveland, OH USA

## Background

Right ventricular dysfunction is a known predictor of mortality in patients with nonischemic cardiomyopathy (NICM), however predictors of right ventricular ejection fraction (RVEF) in this patient population are poorly understood. We sought to identify independent predictors of RVEF, measured using cardiac MRI, in patients with NICM.

## Methods

We retrospectively evaluated 140 consecutive patients with NICM who underwent delayed hyperenhancement cardiac MRI between 2002 and 2009. A Cox proportional hazards model was used to evaluate the association between RVEF and the composite outcome of death, left ventricular assist device implantation, or heart transplantation. Predictors of RVEF were evaluated using a multivariable linear regression model.

## Results

**Figure 1:**
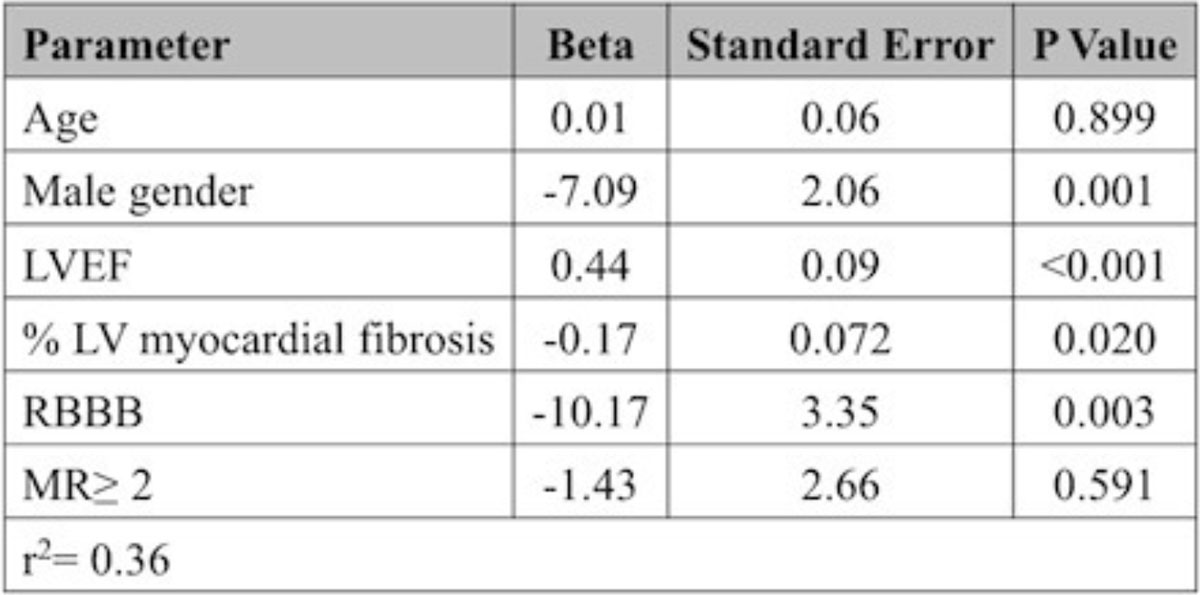
**Predictors of RVEF**.

The study population included 140 patients (mean age 53 ± 17 years, 52% male, mean left ventricular ejection fraction (LVEF) = 33 ± 12%). There were 39 events (death, left ventricular assist device implantation, or heart transplantation) over a median follow-up time of 6.5 years. After adjusting for age, gender, and LVEF, RVEF was a significant predictor of the composite outcome (HR 1.04; p = 0.012). On univariate analysis, male gender, lower LVEF, higher LV myocardial fibrosis, mitral regurgitation (MR) severity ≥ 2 (ß=-6.7; p = 0.013), and presence of right bundle branch block (RBBB) were significantly associated with lower RVEF. After adjusting for age and the aforementioned variables, male gender, LVEF, LV myocardial fibrosis, and presence of RBBB remained significant independent predictors of RVEF, however MR severity was no longer a significant predictor of RVEF (ß=-1.43; p = 0.59). See Figure 1.

## Conclusions

Right ventricular dysfunction has been shown to predict poor outcomes in patients with NICM, but predictors of RVEF in this cohort are not well defined. We found male gender, lower LVEF, higher LV myocardial fibrosis, and the presence of RBBB were independent predictors of lower RVEF. While MR severity was associated with RVEF on univariate analysis, this relationship was not significant independent of LVEF.

